# Educational program for middle-level public health nurses to develop new health services regarding community health needs: protocol for a randomized controlled trial

**DOI:** 10.1186/s12912-018-0287-x

**Published:** 2018-05-08

**Authors:** Kyoko Yoshioka-Maeda, Takafumi Katayama, Misa Shiomi, Noriko Hosoya

**Affiliations:** 1 Department of Health Promotion, National Institute of Public Health, 2-3-6, Minami, Wako-shi, Saitama, Japan; 2 Department of Statistic and Computer Science, School of Nursing, College of Nursing Art and Science, University of Hyogo, 13-71, Kitaoji-cho, Akashi, Hyogo Japan; 3 Department of Community Health Nursing, School of Nursing, College of Nursing Art and Science, University of Hyogo, 13-71, Kitaoji-cho, Akashi, Hyogo Japan; 4 Department of Community Health Nursing, School of Nursing, Chiba Prefectural University of Health Sciences, 2-10-1, Wakaba, Mihama-ku, Chiba-shi, Chiba, Japan

**Keywords:** Public health nursing, Competency, Program development, Randomized controlled trial, Web-based learning

## Abstract

**Background:**

Developing health services is a key strategy for improving the community health provided by public health nurses. However, an effective educational program for improving their skills in planning such services has not been developed. To describe our program and its evaluation protocol for the education of middle-level public health nurses to improve their skills in developing new health services to fulfil community health needs in Japan.

**Methods:**

In this randomized control trial, eligible participants in Japan will be randomly allocated to an intervention group and a control wait-list group. We will provide 8 modules of web-based learning for public health nurses from July to October 2018. To ensure fairness of educational opportunity, the wait-list group will participate in the same program as the intervention group after collection of follow-up data of the intervention group. The primary outcomes will be evaluated using the scale of competency measurement of creativity for public health nurses at baseline, immediately after the intervention. Secondary outcomes will be knowledge and performance regarding program development of public health nurses.

**Discussion:**

This study will enable the analysis of the effects of the educational program on public health nurses for improving their competency to develop new health services for fulfilling community health needs and enriching health care systems.

**Trial registration:**

We registered our study protocol to the University hospital Medical Information Network- Clinical Trials Registry approved by International Committee of Medical Journal Editors (No. UMIN000032176, April, 2018).

## Background

Improving public health is a key to reducing inequalities and the social gradient. Public policy should be developed based on the social determinants of health, and public health care is also required to improve these social determinants [1]. Development, implementation and evaluation of policies and services assist in promoting the health, well-being, and equity of society. The concept of Health in All Policies builds capacity, systems, and resources in the community [2]. In many countries, public health nurses (PHNs) are responsible for promoting and improving community health [3]. Through their daily practices, PHNs identify health issues regarding individuals, families, and communities that require solving by the development of public health policies and services [4]. Filling the gap between community health needs and enriching community health care systems is an important population health management strategy of PHNs [5, 6].

Developing competency to create health services remains one of the most important challenges in public health nursing [7]. In the program planning field, tacit knowledge was found to play a big role, and knowledge translation was a key to developing innovative public health services [8]. We previously demonstrated that PHNs identified the difficulties of clients by focusing on unsolved community health needs. They utilized this evidence as the basis for program development [9]. However, many PHNs felt a lack of competency in planning health programs, and wanted to develop the skills required for it [10, 11]. To increase the capability and confidence of PHNs in planning health services, some educational programs have been conducted [12–15]. These programs chose face-to-face group sessions so PHNs could enrich their knowledge as well as exchange their experiences with each other. However, this educational style is limited regarding the number of participants, owing to time and location constraints.

Web-based learning programs have the potential to overcome such time-location barriers of educational delivery, and they are just as effective as face-to-face education [16]. Trials conducted in Western countries have shown that web-based learning contributes to the efficient improvement of the practical knowledges and skills of PHNs [17–19]. However, the effectiveness of such learning techniques remained unclear because these studies did not compare an intervention group with a control group under a randomized controlled design. Little is known about the effects of web-based learning for PHNs in Asian countries. Yu and Yang reported that most PHNs have a positive attitude toward web-based learning and suggested the necessity of creating a user-friendly web-based learning system [20]. In addition, web-based learning programs lack supervision and communication, and it is difficult to confirm the amount of knowledge that was actually acquired [18]. Therefore, a combination of web-based learning and face-to-face group sessions will be a better way to overcome these disadvantages of web-based learning programs.

Japan has a national license system for PHNs, which contributes to maintaining their quality of care [21]. The Ministry of Health, Labor and Welfare published guidelines for on-the-job training for novice PHNs in 2011 [22]. In particular, it is essential for middle-level PHNs, who are expected to be promoted to a management position in the near future, to develop their skills in program planning [23]. However, middle-level PHNs have not been adequately educated on the theoretical concept and methodology of program planning at their undergraduate level or after employment [24]. In Japan, about 60% of PHNs work at prefectural or municipal governments as public servants [21]. With a decline in the Japanese population, Japanese public governments have promoted personnel downsizing [25]. Under this situation, many PHNs felt busy and faced difficulties in planning new health services [26]. Thus, an ideal educational system for middle-level PHNs would enable learning the theoretical concepts and methodology of health service development at places and times of their choice. Therefore, our aim was to develop a user-friendly educational program for improving the competency of middle-level PHNs’ in creating health services, and evaluating the effectiveness of the program using a randomized controlled design.

### Study objectives and hypotheses

The aim of this study was to evaluate the following: (1) the effectiveness of the newly designed educational program for developing health service-planning skills of PHNs; and (2) identifying the PHNs that will benefit the most from participating in our educational program. In this report we show our research protocol and an overview of our educational program.

The PICO hypothesis of this study is as follows: (1) participants: middle-level PHNs who have worked in public administration; (2) intervention: providing an educational program regarding development of health services; (3) comparison: providing no program, and (4) outcome: improving the participants’ knowledge and performance of health service planning.

## Methods

### Study design and settings

This will be a single-blind and parallel group randomized controlled trial. PHNs will be randomized to either the intervention group or the control group [1:1]. After completing the intervention and collecting the follow-up data, the wait-list control group will have an opportunity to participate in the same educational program as the intervention group.

This study will consist of web-based learning modules and face-to-face group sessions. The study participants will take web-based learning modules at their time and place of choice within Japan. After that, PHNs who want to discuss with other participants about community health needs and how to solve it by developing a new health services will join a face-to-face group session that will be held in Sendai and Kobe, Japan.

### Participants

PHNs must meet the following inclusion criteria: (1) have worked on a full-time basis in public administration, and (2) have experience as a municipal or prefectural PHN of ≥5 years and ≤ 20 years [27]. The exclusion criterion is holding a position above the level of unit chief.

### Sampling, informed consent and randomization

**Fig. 1:**
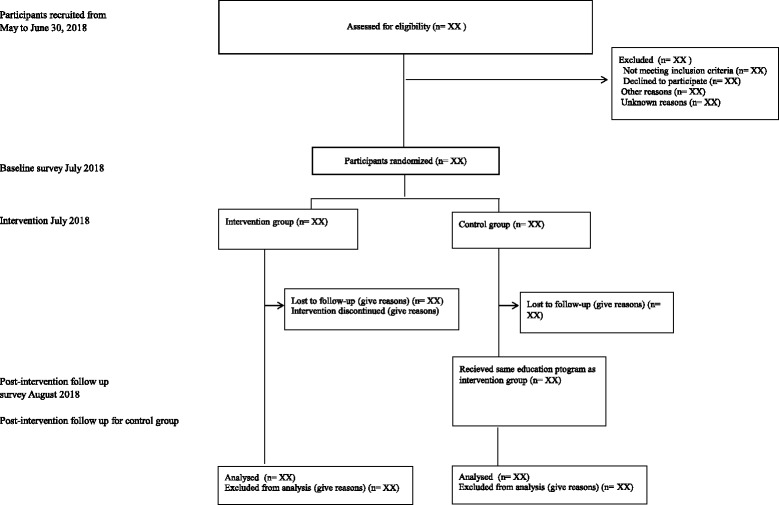
Consolidated Standards of Reporting Trial flow chart

In Japan, the number of PHNs who worked at prefectural or municipal governments in 2014 was 34,500, and one third of them were middle-level PHNs [28]. We will send 48 letters to the head PHNs of prefectural and 128 municipal main government offices in May 2018. We will ask each head PHN to share the information about this study with middle-level PHNs in their institution, and consequently, middle-level PHNs who are interested in this study are expected to access the study home page. Recruitment will be conducted from April to the end of June 2018. Only middle-level PHNs who read the explanation of this study and agree to join it and meet the inclusion criteria will be registered as study participants (Fig. 1). We provided web-based written information about the study, participation and publication of the results. Before study registration, we obtained web-based written informed consent from participants. All researchers will confirm the eligibility of PHNs.

All data were anonymized and do not include any personal information or the name of public administrations. We provided web-based written information about the study and obtained informed consent from participants before study registration. To protect the privacy of the participants, we will prohibit recording of the group sessions and will use nicknames for each participant.

Using a random number table, the statistician who was a co-researcher will allocate participants by equal computer-based randomization to the intervention group and the wait-list control group. To protect privacy, we will not obtain personal information and will not consider the size of each community and local government as adjustment factors. To minimize the difference in the number of participants between the two groups, we will use a permuted-block method.

### Sample size

We calculated that for an effect size of 0.50, 134 PHNs must be analyzed for the evaluation (intervention group: *n* = 67; control group: *n* = 67). This number is assuming a statistical power of 80% and an alpha value of 0.05 by using G*Power 3.1.9.2. [29, 30] We anticipated a drop-out rate of 30%, and hence concluded that 200 participants will be required.

### Minimizing bias

For minimizing bias, the researchers will be blinded regarding the personal information of the participants. The participants will enter their base-line data, follow-up data, and evaluation data of the educational program without involvement of the researchers. The participants will evaluate their competency regarding program planning using the web-based questionnaires that they will fill in by themselves. In addition, to prevent the exchange of information about the educational program between the intervention and the wait-list control group, we will explain the meaning of contamination to all participants in writing and on the homepage. We will send a URL address that can only be opened with a password, and which needs to be accessed for the training materials, to the e-mail addresses of the participants in the intervention group.

### Description of the intervention: Overview of the educational program and its learning objectives

In designing our 3-month educational program for PHNs to acquire skills in health service planning, aimed to solve community health needs identified through reflecting on their daily routine, we will take advantage of the large amount of experience of middle-level PHNs. This is based on previous studies [4, 9, 12, 14, 15, 23, 24], and the adult-learning theory [31] which showed that having a large amount of experience and reflecting on it will contribute to learning.

The main theme and objectives of each module are summarized in Table 1. After watching web-based learning modules 1–3, participants will fill out the worksheet to analyze the association between community health needs and its causes through reflecting on their daily practice. After watching web-based learning sessions 4–8, participants will write a proposal of a new health service based on community health needs that they identified.

**Table 1:** Title of web-based learning modules, optional group session, and their objectives

	Title of module		Objectives
Web-based learning modules	1. Recall your great experiencing as a public health nurse	1	To understand the theoretical concept of program development, to efficiently solve the health issues of individuals and the community.
		2	To rediscover the role of PHNs through reflection of their own practice.
		3	To build motivation of participation.
	2. Finding time to think about program planning within your busy work schedule	1	To understand the necessity of maximizing the efficiency of their daily practices.
		2	To understand the needs of preventive intervention by PHNs.
	3. Identifying community health needs through analyzing the people who require a large amount of the time and help of PHNs	1	To clarify the health needs of the people who require a large amount of time and help from the PHNs.
		2	To analyze the causal relationships of the community health need.
	4. Organizing the evidences to confirm the necessity of program development	1	To understand the evidences to confirm the necessity of program development and how to collect such evidence.
	5. Developing a partnership with your colleagues and collaborators for promoting a program planning	1	To understand the key community stakeholders and collaborators of program planning.
		2	To share the community agenda and to build a consensus with the collaborators.
	6. Breaking the difficulty of obtaining budget	1	To understand the budget for developing a new program.
		2	To understand the necessity in cost-benefit-analysis of program planning.
	7. Common mistakes for analyzing a communith health need	1	To understand the common mistakes for analyzing a community health need.
	8. Common mistakes for developing a proposal of a new health program	1	To understand the common mistakes for developing a proposal of a new health program.
Optional group session	1. Identifying community health needs and developing a proposal of a new health program	1	Through face-to-face group sessions, participants will analyze and identify community health needs.
		2	Identifying the necessity of evidence which will support the existence of community health needs, and discuss about ways to fulfill them.
		3	Creating their proposal of a new health program: methods, expected effects, plan of evaluation indicators.

After watching 8 web-based modules, we will have an optional group session for the PHNs who want to discuss with other participants about community health needs and developing a proposal of a new health program. Each group will consist of 5 or 6 participants, to enable in-depth discussions. To decrease dropout rate, group session is an optional module.

After developing our educational program, we asked four middle-level PHNs and one professor who supervises PHNs working in prefectures and municipalities to check the contents of it as a pre-test. In accordance with their comments, we revised the explanation and confirmed that the contents of the program will cover the main skills of program planning that the PHNs should acquire.

### Wait-list control group

To ensure fairness in educational opportunity, the control wait-list group will participate in the same educational program as the intervention group after collection of the follow-up data from the intervention group.

### Data collection

After registration of the eligible participants, we will collect individual data at baseline, immediately after the intervention. At baseline, all participants will fill in a web-based questionnaire including demographic information, such as age, gender, education level, position, population size of their municipality, number of residents per PHN in their municipality, their affiliation, previous experience in developing or participating in program development, existence of colleagues or bosses who actively work on program development, and daily practices that will help to build their skills in program planning. We will send the news letters for all participants to prevent drop out. This research was not harmful intervention, so we do not have data monitoring committee.

### Outcomes and assessments

#### Competencies of program planning

The primary outcome, which is competency in program planning, will be measured using the Competency Measurement of Creativity for PHNs (CMC) (Shiomi et al., 2010). The CMC includes 16 self-reported items. The first 3 items measure the competency of PHNs in identifying the necessity of new health services. The next 9 items evaluate skills in the promotion and realization of a service plan. The last 4 items evaluate skills in collaborating with others towards developing a health service. Items are scored on a six-point Likert scale, as follows: 0, not applicable; 1, 20% applicable; 2, 40% applicable; 3, 60% applicable; 4, 80% applicable; and 5, 100% applicable.

#### Knowledge and performance regarding health service development

We will evaluate the educational program using 26 questions regarding the knowledge and practice of PHNs on health service development, at baseline and after the intervention. The participant will fill the questionnaires from 0 (very poor) to 10 (very good) points.

#### Validity and reliability

The scale used to measure primary outcomes used in this study has been confirmed for its reliability and validity, and have been utilized widely in Japan. To ensure data quality, we will collect all data through the original web-site for this study. Additionally, to prevent drop-out from the study, we will send e-mails to all participants and remind them to fill out the web-based follow-up questionnaire.

#### Statistical analysis

Intention-to treat analysis will be utilized to assess the main effect of our intervention. We will calculate the initial drop-out rate as 30%, which will affect the sample size. Even if this rate exceeds 30%, we will not perform an additional recruitment of participants. We will compare the baseline characteristics of the intervention and control group using the *t*-test, and chi-square/Fisher exact test. In addition, ANOVA will be performed to identify factors affecting CMC, and knowledge and behavior regarding program development. To confirm significant improvements in competency, logistic regression analysis will be conducted. PHNs will also be analyzed in groups by years of experiences as PHNs, to identify the group that will benefit the most from participating in our educational program.

#### Ethical considerations

The Institutional Review Board of Nursing Research in Tokyo Medical University approved this study protocol on March 2, 2017 (ID: 28–11). The trial was registered in the University hospital Medical Information Network- Clinical Trials Registry (UMIN-CTR) which was acceptable registry of International Committee of Medical Journal Editors (No. UMIN000032176, April, 2018). The Institutional Review Board and the UMIN-CTR monitored the progress of this study every 6 months. We provided web-based written information about the study, participation and publication of the results. We obtained web-based written informed consent from participants before study registration. All participants also agreed that we will publish the results of this study.

## Discussion

This randomized controlled trial was carefully designed, and aimed to examine the effects of an educational program on improvement of the knowledge and competency of middle-level PHNs regarding health service planning to fulfil community health needs. The strengths of our education program are: (1) developing a user-friendly educational program that combined web-based modules and face-to-face group sessions, (2) participants will be recruited nationwide using clear criteria and will be representative of PHNs in Japan, (3) the program ensures fairness of educational opportunity to participants in the control group, who will have a chance to take the same educational program as the intervention group, and (4) primary outcome measures, including the competency of PHNs, are validated and evaluated using a standardized scale.

We first developed an educational program for middle-level PHNs comprising a combination of web-based modules and face-to-face group sessions. Previous studies only used web-delivered educational modules [32], educational workshops [12], or face-to-face group sessions with homework [13, 15]. Web-based learning methods can overcome time-location restrictions, and will contribute to the nationwide recruitment of subjects, resulting in a sample that is representative of Japan. If our results show a positive effect, our program is expected to contribute to the development of program planning skills of middle-level PHNs, which will be a key to improving health care systems for fulfilling community health needs.

Second, we will provide the same educational opportunity to the control group, which will contribute to identify the effectiveness of our educational program. Assuring equal chance will contribute to prevent the drop-out of participants in the control group, and to accurately understand the effects of the intervention. From the results, we will be able to determine the group of middle-level PHNs that will benefit the most from the program, and the type of skills that can be developed through the program. Using validated and standardized scales will clearly show the effects of the intervention.

This study has a few limitations. First, the drop-out rate will be high and the number of participants will be limited. Because it is common for PHNs to attended on-the-job training, which is based on an order from their boss as part of their work on weekdays in Japan. Our study is off the job training on weekends. Despite of sending letters of recruitment throughout Japan, the PHN who have high motivation will complete all modules. This will be a potential factor for selection bias in the present study.

Second, contamination of the intervention will occur in the control group. We will explain that sharing of the contents of the intervention among study participants is strictly prohibited. However, we will not be able to confirm whether the participants actually follow our instructions or not. Because of the protection of privacy, we will not collect the names of the communities in which the PHNs are working. Therefore, we will not be able to identify PHNs working in the same organization and put them into different educational group.

Despite of these limitations, this randomized controlled study will show the effects of an educational program aimed to improve the competency of middle-level PHNs to develop new health services for solving community health needs in Japan. Through collection of data over one year, we will confirm whether our user-friendly educational program will be useful for improving program planning competency of middle-level PHNs.

## Conclusions

This study will enable the analysis of the effects of the educational program on public health nurses for improving their competency to develop new health services for fulfilling community health needs and enriching health care systems. We will analyze how to apply their off-the-job training for filling the gap between community health needs and health care systems in each community.

## Abbreviations

PHN: Public health nurse
UMIN-CTR: University hospital Medical Information Network- Clinical Trials Registry
